# Analyzing when parental warmth but without parental strictness leads to more adolescent empathy and self-concept: Evidence from Spanish homes

**DOI:** 10.3389/fpsyg.2022.1060821

**Published:** 2022-12-05

**Authors:** Maria C. Fuentes, Oscar F. Garcia, Marta Alcaide, Rafael Garcia-Ros, Fernando Garcia

**Affiliations:** ^1^Department of Methodology, Faculty of Psychology, University of Valencia, Valencia, Spain; ^2^Department of Developmental and Educational Psychology, University of Valencia, Valencia, Spain

**Keywords:** parenting styles, empathy, self-concept, perspective taking, adolescents

## Abstract

**Introduction:**

Classical research mainly conducted with European-American families has identified the combination of warmth and strictness (authoritative style) as the parenting always associated with the highest scores on developmental outcomes. Additionally, despite the benefits of empathy for prosocial behaviors and protection against antisocial behaviors, most research has considered the contribution of specific practices (e.g., reasoning or power assertion), but not so much the parenting styles. Similarly, family studies tend to study the relationship between parenting and global self-perceptions (self-esteem), but not so much those of each dimension (self-concept).

**Methods:**

In the present study, 600 Spanish adolescents from 12 to 17 years old (*M* = 15.25, *SD* = 2.01) were classified within one of the four household typologies (i.e., authoritative, indulgent, authoritarian, or neglectful). Adolescent developmental outcomes were cognitive empathy (adopting perspectives and emotional understanding), emotional empathy (empathic stress and empathic happiness), and self-concept (academic, social, emotional, family and physical).

**Results:**

The results showed that the indulgent parenting (warmth but not strictness) was related to equal or even better empathy and self-concept than the authoritative style (warmth and strictness), whereas non-warm parenting (authoritarian and neglectful) was consistently associated with poor results.

**Discussion:**

Overall, the present findings seriously question that parental strictness combined with parental warmth (authoritative style) is always the parenting style related to the greatest outcomes. By contrast, it seems that reasoning, warmth and involvement, without strictness (indulgent parenting) help adolescents to achieve a good orientation toward others in terms of cognitive and affective empathy and a good self-evaluation in terms of self-concept.

## Introduction

The internalization of social values is usually defined as “taking over the values and attitudes of society as one’s own so that socially acceptable behavior is motivated not by anticipation of external consequences but by intrinsic or internal factors” (Grusec and Goodnow, 1994, p. 64). For many years, scholars have discussed how parents can help children to acquire a set of social values to develop the capacity to take the perspective of others, and self-regulatory abilities, including responsibility, psychosocial maturity, and an adequate sense of self in their children (Maccoby and Martin, 1983; Grusec and Goodnow, 1994; Grusec et al., 2017).

Parents can encourage or damage the child’s capacity to make inferences about how others feel (i.e., empathy) as well as they can foster or harm the child in the construction of his/her portrait as a valuable individual with good qualities (i.e., self-concept; Baumrind, 1978; Pinquart and Gerke, 2019; Martínez et al., 2021). Overall, empathy includes the cognitive trait (e.g., capacity to explicitly infer mental states in others) as well as the affective trait (e.g., capacity to share and understand the internal state of others; Jolliffe and Farrington, 2006; Farrant et al., 2012; Yu and Chou, 2018). Self-concept refers to the evaluative component of self-perceptions, which includes a global dimension, but also different dimensions related to each other (e.g., emotional and social self-concept; Harter, 1988; Garcia F. et al., 2018; Chen et al., 2020). Empathy and self-concept, both positively correlated, are strongly linked to prosocial behaviors (Laible et al., 2004; Ramirez-Jimenez and Serra-Desfilis, 2020). By contrast, antisocial behaviors are associated with lack of empathy and self-concept (Gracia et al., 2008; Lila et al., 2013; Ruiz-Hernández et al., 2021).

Despite variations in the way academics have studied parental socialization over time, researchers agree on identifying two major dimensions (Sears et al., 1957; Baumrind, 1967; Maccoby and Martin, 1983), warmth and strictness, also referred to as responsiveness and demandingness in empirical research (Lamborn et al., 1991; Steinberg et al., 1994), or acceptance/involvement and strictness/imposition (Martínez et al., 2012, 2017). For example, earlier scholars were already using labels such as warmth, assurance or love, and domination, and labels such as firm discipline or control, with similar meanings to the warmth and strictness dimensions (Symonds, 1939; Baldwin, 1955; Schaefer, 1959; Becker and Krug, 1964). The two parental dimensions are theoretically orthogonal (i.e., unrelated) constructs reflecting two persistent patterns of parental behavior in children’s socialization process that organize the different parenting practices (Darling and Steinberg, 1993; Garcia et al., 2015; Ibabe, 2019; Tur-Porcar et al., 2019).

Specifically, the warmth dimension refers to the degree to which parents are emotionally involved in their children’s socialization, showing them warmth, affection, and support, and using dialog and reasoning as main parenting strategies to modify their maladjusted behavior (Maccoby and Martin, 1983; Lamborn et al., 1991; Gimenez-Serrano et al., 2021, 2022). The strictness dimension refers to the extent to which the parental behavior is characterized by firmness and strict discipline, using parenting practices such as control, scolding, or spanking to modify their children’s maladjusted behavior and clearly set limits on their conduct (Maccoby and Martin, 1983; Axpe et al., 2019; Gimenez-Serrano et al., 2021). Four parenting styles are defined by the combinations of the two main parenting dimensions: The authoritative style (high warmth and high strictness), the indulgent style (high warmth and low strictness), the authoritarian style (low warmth and high strictness), and the neglectful style (low warmth and low strictness).

Scholars as Baumrind and Lewis have discussed how parents can favor an effective socialization, usually identify in terms of consideration for others and good personal competence. Lewis (1981) conjectured that parental warmth, regardless of parental strictness, promotes an effective socialization, despite empirical evidence revealed the benefits of authoritative parenting to help children to achieve good prosocial behavior and internalization of the social norms (Baumrind, 1967; Baumrind and Black, 1967; Baumrind, 1971), but also confidence in one-self and good self-esteem (Coopersmith, 1967). Nevertheless, Lewis (1981) argued that children from authoritative homes internalize social norms may be due to the parental warmth rather than by parental strictness. Specifically, based on attribution theory, Lewis (1981) suggests that the *least* salient external control (i.e., parental warmth) might be sufficient to elicit the greatest child internalization of the social norms whereas those more salient external controls (i.e., parental strictness) might be superfluous, so its deletion from authoritative parenting package is not associated with less well-socialized behavior.

As response to Lewis, Baumrind (1983) argued that both warmth and strictness parental ingredients are independently (only appears joined in the authoritative parenting) and she emphasized the relevance of parental strictness as an essential ingredient of the authoritative package. Baumrind (1983) noted that warmth (higher parent–child communication, and nurturance) and strictness (greater control and maturity demands) should appear simultaneously to achieve a well-socialized child. Therefore, according to Baumrind (1983), it is not enough with the parental warmth (e.g., parent–child communication) regardless strictness, contrary to Lewis statements. Instead, Baumrind (1983) pointed out that strictness is not an incidental component of authoritative parenting style, rather than its main component that defined an optimal parenting. Specifically, Baumrind explained the widely benefits of parental strictness, except for when punishment is cruel or noncontingent (agree with Lewis). Additionally, benefits are greater for children when parents combined parental control and high maturity demands (i.e., strictness), and they are responsive and sensitive (i.e., warmth). Data from the so-called three parenting styles (i.e., authoritative, authoritarian, and permissive) were widely supported by empirical evidence (see Darling and Steinberg, 1993).

The theoretical parenting framework also comprises the consequences of the different child-rearing patterns on child and adolescent competence and adjustment (Darling and Steinberg, 1993; Villarejo et al., 2020). One of the most consistent results, based on studies mostly with Anglo-Saxon samples from middle-class families, shows that high parental warmth along with high parental strictness (i.e., authoritative style) provides the greatest psychosocial benefits to children (Baumrind, 1967; Baumrind, 1971; Lamborn et al., 1991; Steinberg et al., 1994). For instance, the authoritative style is related to the highest scores on several criteria, such as psychological adjustment (Kritzas and Grobler, 2005), academic achievement (Im-Bolter et al., 2013), adaptive strategies (Aunola et al., 2000), and protection from deviance such as antisocial behavior and drug use (Montgomery et al., 2008; Hoffmann and Bahr, 2014). However, some studies carried out in the US with ethnic minority groups, including Asian-American (Steinberg et al., 1992; Chao, 2001) or African-Americans (Baumrind, 1972; Pittman and Chase-Lansdale, 2001), as well as some evidence from Asian and Middle Eastern societies (Dwairy and Achoui, 2006; Dwairy et al., 2006b,c), raise doubts about the authoritative style as the best parenting strategy to provide the greatest benefits for adolescents in all cultural and ethnic contexts. Instead, they find that authoritarian parenting (warmth but not strictness) is related to some optimal child and adolescent outcomes. Specifically, it is argued that in these cultural contexts children and adolescents could interpret the authoritarian parenting as a form of protection and caring (Leung and Shek, 2020; Martínez et al., 2021).

These discrepancies in findings from studies examining the relationship between child-rearing patterns and adolescent competence and adjustment seem to suggest variations in optimal parenting depending on the cultural and social context where parental socialization is examined (Darling and Steinberg, 1993; Pinquart and Kauser, 2018; Pinquart and Gerke, 2019; Garcia, Fuentes, et al., 2020). In addition, recent family studies are examining parenting and its impact on child competence across different cultural contexts, including Western societies (Wade et al., 2018) as well as Eastern societies (Yeung, 2021). Interestingly, a recent parenting study (Garcia et al., 2019) suggests that three parenting stages (authoritarian, authoritative and indulgent) for the optimal parenting might concur in the Digital Society at the same time in different environments, contexts, and cultures, extending previous evidence based on the traditional paradigm with only two stages (i.e., authoritarian and authoritative parenting styles). The indulgent parenting (i.e., warmth without strictness) is often related to optimal competence and adjustment, according to studies mostly conducted with families in European (Rodrigues et al., 2013; Calafat et al., 2014; Garcia et al., 2019) and South-American countries (Martínez et al., 2007; Martínez and Garcia, 2008; Garcia et al., 2019). The third parenting stage (i.e., the indulgent style) is related to the same or even better scores than the authoritative style on different indicators of adolescent competence such as psychological adjustment (Fuentes et al., 2015b), self-concept (Calafat et al., 2014; Perez-Gramaje et al., 2020), internalization of social values (Martínez and Garcia, 2007) and environmental values (Queiroz et al., 2020), connectedness to nature (Musitu-Ferrer et al., 2019a), positive attitude toward institutional authority (Martinez-Ferrer et al., 2018) or school competence (Fuentes et al., 2015c). In addition, indulgent parenting provides broad benefits in terms of protection against alcohol and other drugs (Fuentes et al., 2015a; Garcia, Serra, et al., 2020), traditional bullying and cyberbullying victimization (Martínez et al., 2019), problematic use of social networking sites (Martinez-Ferrer et al., 2018), child-to-parent violence (Suárez-Relinque et al., 2019) or behavioral problems (Martínez et al., 2013). Some recent studies extend the benefits of indulgent parenting beyond adolescence (Garcia O.F. et al. 2018; Garcia and Serra, 2019; Martinez-Escudero et al., 2020; Garcia et al., 2021).

### The present study

The aim of this study was to examine which parenting style (indulgent, authoritarian, authoritarian, or neglectful) is associated with greater benefits in achieving adolescents who can take the perspective of others and have an adequate sense of self. Specifically, it was examined (i) cognitive empathy (adopting perspectives, emotional understanding), emotional empathy (empathic stress and empathic happiness), and (ii) self-concept (academic, social, emotional, family, and physical). The process of internalization of self-transcendence values involves socially-focused motivations (Sortheix and Schwartz, 2017), emphasizing the positive effects on others of fostering a child’s feelings of empathy and consideration for others (Hoffman, 1970; Lewis, 1981; Baumrind, 1983).

Nevertheless, although social values help children to be able to take the perspective of others, less is known about the impact of parenting on children’s empathy, which might be an important expression of social values that emphasize accepting others as equals and having concern for their welfare (Caprara et al., 2012). Most of the available evidence stems from studies conducted in Europe (Carlo et al., 2011), the United States (Hoffman and Saltzstein, 1967) or Asia (Yan et al., 2017) that examine parenting practices rather than parenting styles, with some unexpected findings (Hoffman and Saltzstein, 1967; Krevans and Gibbs, 1996; Helwig et al., 2014; Llorca-Mestre et al., 2017; Boele et al., 2019). For example, Hoffman and Saltzstein (1967) examined three discipline practices: Power assertion (the parent capitalizes on his power and authority over the child), love withdrawal (direct but nonphysical expressions of anger or disapproval), and inductive reasoning (explanations of parents about the consequences of the child’s action for others). Among middle-class families, children with the best competence oriented toward the feelings of others are those whose parents use inductive reasoning and less power assertion, although, unexpectedly, among lower-class families, competence toward others is related to love withdrawal, but unrelated to inductive reasoning. Krevans and Gibbs (1996) found that children whose parents used inductive reasoning reported the highest empathic competence, but contrary to the authors’ expectations, parents’ use of statements of disappointment was strongly related to children’s empathic abilities. As Boele and colleagues noted in their recent literature review (2019), it is unclear how the parent–child relationship could be associated with empathy outcomes, partly because theoretical and methodological weaknesses in the focus of parental practices make it difficult to find conclusive answers (Boele et al., 2019).

Specifically, common parental practices in empathy studies (e.g., reasoning induction, love withdrawal, psychological control, or power assertion) are usually captured in isolation rather than in the general context of the two orthogonal dimensions (i.e., unrelated; Hoffman and Saltzstein, 1967; Krevans and Gibbs, 1996; Helwig et al., 2014; Boele et al., 2019). By contrast, parenting styles also includes the different practices used by parents but classified and ordered based on two main dimensions (Maccoby and Martin, 1983; Darling and Steinberg, 1993). The study of parenting within the context of parenting styles might add more clearly evidence. The empirical findings about the consequences of parenting practices for children could be different depending on if the parental practices are examined alone or at the same time main time ordered in the two main axis (i.e., warmth and strictness). Baumrind noted (1983) that the negative effects of power assertion (i.e., greater strictness) for moral development identified by Hoffman and Saltzstein (1967), despite power assertion is one of the main components of authoritative parenting, could be explained by its effect is not studied at the same time with inductive reasoning (i.e., greater warmth) within the more general climate represented by the parenting style. Specifically, Baumrind (1983) argued that “the use of reasoning accompanied by power assertion should be more effective with young children than reasoning alone” (p. 141). The benefits of power assertion, according to Baumrind (1983), may be due to the “the use of a reason accompanied by a display of power also conveys to the child that adherence to a rule of right conduct is independent of the presence of the parent” (p. 141).

Additionally, the cultural contexts seem to be crucial to explain why the same parenting seems to be related to different consequences for child and adolescent development, including for empathy (Darling and Steinberg, 1993; Krevans and Gibbs, 1996; Carlo et al., 2011; Helwig et al., 2014; Boele et al., 2019). As Helwig et al., (2014) p. 19, “we know little about children’s comprehension of the processes by which different discipline methods are judged to be effective, and virtually nothing about their understanding of the psychological consequences of different types of discipline.” In the few previous studies examining the four parenting styles and empathy, an important limitation is that they captured empathy as a global dimension, but without adopting a multidimensional perspective (Garcia and Serra, 2019; Garcia et al., 2021).

Moreover, self-esteem is usually examined in parenting styles self as a global dimension (e.g., Pinquart and Gerke, 2019), but not examining specifically each dimension of self, such as emotional or physical self-concept. The impact of parenting on adolescent psychosocial competence is consistently identified in several studies with adolescents examining different indicators (Garcia et al., 2019; Gallarin et al., 2021; Ridao et al., 2021). In order to explain how parents can contribute to foster adolescent psychosocial competence, self-concept could be especially relevant in part because it reflects the subjective evaluation of adolescent self-competence (Lord et al., 1994; Martínez et al., 2021). Findings from classical studies, mainly conducted with middle-class families from United States, revealed that parental warmth favors greater self-esteem but only when is accompanied by strictness (Coopersmith, 1967; Barber et al., 1992; Pinquart and Gerke, 2019). Adolescents raised in homes in which parents use strictness and imposition, combined with the use of warmth, tend to report greater self-concept than their peers from other households (Lamborn et al., 1991; Darling and Steinberg, 1993; Pinquart and Gerke, 2019) and, at the same time, they are protected against deviance in the social realm. By contrast, adolescents from indulgent homes tend to benefit of greater warmth for their self-concept (as those from authoritative homes), but their lack of parental strictness harm them in the realm of social norms. However, some recent studies revealed adolescents with greater self-concept are those whose parents are warm and involved, while parental strictness could be unnecessary or even harmful for adolescent self-concept (Riquelme et al., 2018; Queiroz et al., 2020).

Sex and age have been considered important variables to understand the consequences of parenting on child and adolescent development. The optimal parenting could be not always the same depending on child sex. Mainly based on studies on parenting practices, some gender scholars have suggested that the consequences of parenting could be distinct for daughters and sons. For example, parental warmth was identified as protective factor against child-to parent violence in daughters but not in sons (Beckmann et al., 2021) and parenting need support (great warmth), which was reported by participants about their mothers, predicted changes in empathic concern in daughters only (Miklikowska et al., 2011). Similarly, unsupervised time at home alone (low strictness) was related to greater smoking only for daughters (Griffin et al., 2000), and poor parental discipline (low strictness) was associated with aggression and other externalizing behavioral problems in sons, but not in daughters (Hosokawa and Katsura, 2019). On the opposite side, other studies not found differences in the impact of parenting depending on child sex (Perez-Gramaje et al., 2020; Queiroz et al., 2020; Gimenez-Serrano et al., 2021). Additionally, some studies test if the consequences of parenting could change depending on child age. Overall, findings are mixed. Some small variations in the impact of parenting depending on child age were identified, for example, child age moderated the relation between negative affect and supportive-positive parenting (i.e., great warmth), but not harsh-negative parenting (i.e., great strictness; Rueger et al., 2011). On the opposite side, other studies found that the impact of parenting is the same regardless child age (Riquelme et al., 2018; Moreno-Ruiz et al., 2019). Therefore, parenting studies usually includes in the design sex and age as factors (i.e., independent variables) to test the possible interaction effect between parenting and child sex, as well as between parenting and child age.

Based on previous studies, we would expect the parenting style characterized mainly by parental warmth, but not by parental strictness (i.e., the indulgent style), to be related to equal or even higher cognitive and emotional empathy and greater self-concept than the parenting style characterized mainly by parental warmth and strictness (i.e., the authoritative style), whereas both parenting styles characterized by lack of warmth (i.e., authoritarian and neglectful) would be related to poor empathic competence and self-concept.

## Materials and methods

### Sample and procedure

A minimum sample size of 600 participants was estimated in an *a priori* power analysis in order to carry out the present study with a statistical power of.95 (the conventional, *α* = 0.05, *β* = 0.05, 1 − β = 0.95) medium-small effect size (*f* = 0.17, Cohen, 1977) in a univariate *F*-test among the four parenting style groups (Pérez et al., 1999; Garcia et al., 2008; Faul et al., 2009). Based on the complete list of high schools from a metropolitan area in the East of Spain with a population of about one million inhabitants, six schools were randomly selected to participate (Garcia, Serra, et al., 2020). The headmaster of each school was contacted (one of them declined to participate). The students who freely chose to participate had previously received their parents’ permission to complete the questionnaires in one class period (93% response rate). Respondents’ anonymity and confidentiality were guaranteed. All respondents were Spanish as were their parents and grandparents. Participants have a similar socioeconomic status; they were from middle-class families. Finally, participants in the study consisted of 586 adolescents, 329 females (56.1%) and 257 males (43.9%), from 12 to 17 years old (*M* = 15.25, *SD* = 2.01). A post-hoc sensitivity analysis for this sample size indicated that the expected medium-small effect size could be detected, *f* = 0.171 (Cohen, 1977), with a power of 0.95 (*α* = 0.05, *β* = 0.05, 1 − *β* = 0.95) (Gracia et al., 1995; Faul et al., 2009). The research protocol of present study conforms to recognized standards from Declaration of Helsinki and it was approved by the research ethics committee of the Program for the Promotion of Scientific Research, Technological Development, and Innovation of the Valencian Community, which supported this research.

### Measures

Parenting styles were measured with the Parental Socialization Scale, *ESPA29* (Musitu and Garcia, 2001), based on the classical two-dimensional theoretical model of parental socialization (Maccoby and Martin, 1983; Darling and Steinberg, 1993). Warmth was measured with the acceptance/involvement ESPA-29 measure, and strictness was captured with the strictness/imposition ESPA-29 measure. The questionnaire consists of 212 items that follow a contextual and situational perspective (Smetana, 1988; Darling and Steinberg, 1993). Adolescents responded on a 4-point scale (1 = never, 4 = always) to rate the frequency with which both their father and mother (considered separately) employ practices in 29 representative scenes from daily family life in Western culture. Thirteen of these 29 scenes refer to adolescent compliance situations (e.g., “If somebody comes over to visit and I behave nicely”), and 16 refer to adolescent noncompliance situations (e.g., “If I do not study or I do not want to do the homework from school”). In each of the compliance situations, parenting practices of warmth (“he/she shows affection”) and unresponsiveness (“he/she seems indifferent”) are rated by adolescents. In the noncompliance situations, parenting practices of reasoning (“he/she talks to me”), detachment (“it’s all the same to him/her”), verbal scolding (“he/she scolds me”), physical punishment (“he/she spanks me”), and revoking privileges (“he/she takes something away from me”) are rated by adolescents.

The factorial structure of this instrument has been confirmed in various studies conducted in different countries such as Spain, Portugal, Brazil (Martinez et al., 2019), or the United States (Martínez et al., 2017), as well as its factorial invariance across demographic variables such as age and sex (Martínez et al., 2012). Moreover, the orthogonality of the two major measures has been supported (Garcia and Gracia, 2014; Garcia et al., 2015). The different parental practices captured by the ESPA-29 are examined within the two main parenting dimensions (i.e., acceptance/involvement and strictness/imposition). The family score on acceptance/involvement was obtained by averaging the responses for warmth, reasoning, unresponsiveness, and detachment (on the last two subscales, the responses were reversed because they were negatively related to the dimension). The family score on strictness/imposition was obtained by averaging the responses for verbal scolding, physical punishment, and revoking privileges. The ESPA29 subscales offer an accurate and reliable measures in which the adolescent gives 212 responses (106 for the father and 106 for the mother, both considered separately). Great reliability for the different subscales is usually reported in studies with adolescents in which parenting is measured by the ESPA-29 (Martínez and Garcia, 2007; Fuentes et al., 2015b; Martínez et al., 2017; Garcia et al., 2019; Martinez et al., 2019, 2020). The ESPA-29 offers a very reliable measure of parenting which is usually used not only in research (del Milagro Aymerich et al., 2018; Martinez-Ferrer et al., 2018) but also for practice (e.g., clinical and forensic psychology; Castaneda et al., 2012). The alpha values tend to be equal to or even higher than.90 for all subscales according to previous studies with adolescents (Martinez-Ferrer et al., 2019; Musitu-Ferrer et al., 2019a; León-Moreno et al., 2020). Ratings for mother and father were averaged as in some studies mainly focused on identify the best parenting regardless of who is the main caregiver (see Lamborn et al., 1991). Both family indices range from 1 to 4 points, and so higher scores represent higher levels of acceptance/involvement and strictness/imposition. Cronbach’s alphas for the subscales were: Warmth, 0.96, unresponsiveness, 0.95, reasoning, 0.96, detachment, 0.93, verbal scolding, 0.95, physical punishment, 0.96, and revoking privileges, 0.97. Cronbach’s alphas for the two major dimensions were: Acceptance/involvement, 0.98, and strictness/imposition, 0.97.

Cognitive and emotional empathy were measured using the Cognitive and Affective Empathy Scale, *TECA* (Fernández-Pinto et al., 2008; Lopez-Perez et al., 2018), based on the classical theoretical framework distinguishing the emotional and cognitive features of empathy (Davis, 1983). Cognitive empathy was captured with the *TECA* subscales: Adopting perspectives (e.g., “I try to take into account all viewpoints before making decisions”) and emotional understanding (e.g., “I realize when someone tries to hide his/her feelings”). Adopting perspectives refers to the intellectual or imaginative capacity to put oneself in the place of another, and the emotional understanding dimension refers to the ability to recognize and understand situations. Emotional empathy was captured with the two *TECA* subscales: Empathic stress (e.g., reversed item, “I consider myself a cold person because I do not get excited easily”) and empathic happiness (“I feel good if others have fun”). Empathic Stress is the ability to share someone else’s negative emotions, and empathic happiness refers to the ability to share another person’s positive emotions (Gorostiaga et al., 2014). The *TECA* four-factor structure was confirmed with confirmatory factor analysis. Additionally, *TECA* showed adequate reliability and validity (Fernández-Pinto et al., 2008; Gorostiaga et al., 2014). Cronbach’s alpha for each subscale was: Adopting perspectives, 0.75, emotional understanding, 0.71, empathic stress, 0.70, and empathic happiness, 0.79.

Self-concept was measured using the Multidimensional Self-Concept Scale, *AF5* (Garcia and Musitu, 1999), based on Shavelson’s multidimensional and hierarchical theoretical framework (Shavelson et al., 1976; Byrne and Shavelson, 1996). It consists of 30 items, with a response scale ranging from 1 (“strongly disagree”) to 9 (“strongly agree”). It assesses five self-concept domains, with 6 items for each dimension: Academic (e.g., “I do my homework well “), social (e.g., “I make friends easily”), emotional (e.g., reversed item, “I am afraid of some things”), family (e.g., “My parents give me a lot of confidence”), and physical (e.g., “I take good care of my physical health”). Higher scores represent a greater sense of self-concept in any of the dimensions.

The *AF5* five-dimensional factor structure was confirmed using both exploratory and confirmatory factor analysis in studies conducted in several countries such as Spain (Murgui et al., 2012; Fuentes et al., 2020), Chile (Garcia et al., 2011), Brazil (Garcia F. et al., 2018), Portugal (Garcia et al., 2006) or United States (Garcia et al., 2013) and China (Chen et al., 2020), as well as its factorial invariance across age and sex (Fuentes et al., 2011). Several studies have also found that negatively worded items showed no method effects (Tomás and Oliver, 2004; Garcia et al., 2011, 2013). Cronbach’s alpha for each subscale was: Academic, 0.90, social, 0.79, emotional, 0.80, family, 0.96, and physical, 0.81.

### Plan of analysis

First, families were classified according to their parenting style. For this purpose, the sample was dichotomized based on the median-split procedure, considering the scores on the acceptance/involvement and strictness/imposition dimensions simultaneously (Chao, 2001), and also taking into account the children’s sex and age (Musitu and Garcia, 2001). Thus, authoritative families were those who scored above the median on both dimensions, indulgent families scored above the median on acceptance/involvement and below it on strictness/imposition, authoritarian families scored below the median on acceptance/involvement and above it on strictness/imposition, and, finally, neglectful families scored below the median on both dimensions (Lamborn et al., 1991; Steinberg et al., 1991). The split procedure is frequently used in studies about parenting styles to assign families to the parenting groups (i.e., authoritative, indulgent, authoritarian and neglectful) based on the responses in the two main parenting dimensions, i.e., warmth and strictness (Lamborn et al., 1991; Steinberg et al., 1994; Garcia and Gracia, 2009; Garcia and Serra, 2019; Garcia, Fuentes, et al., 2020; Queiroz et al., 2020; Gimenez-Serrano et al., 2021). The use of the median-split procedure to classify families into the parenting styles (i.e., authoritative, indulgent, authoritarian and neglectful), rather than assigning according to predetermined cutoffs, provides a categorization of families that is sample-specific. For instance, families in the authoritarian parenting style are indeed relatively more authoritarian (i.e., use more strictness/imposition and less acceptance/involvement) than the other families in the sample, although we do not know if the families labeled as “authoritarian” would be considered “authoritarian” within a different population. Thus, it should be note that the categorization of families as one type or another, compared to the other families, is done for heuristic, not diagnostic, purposes (see Lamborn et al., 1991, p. 1053; Queiroz et al., 2020, p. 5; Garcia and Serra, 2019, p. 6).

Second, a multivariate factorial design (MANOVA, 4 × 2 × 2) with each set of criteria (empathy and self-concept) was performed considering the parenting style (authoritative, indulgent, authoritarian, and neglectful), the children’s sex (male and female), and the children’s age (12–14 years and 15–17 years) as independent variables, in order to test possible interaction effects. Age groups are the same as those from other parenting studies (e.g., Martínez et al., 2019). After that, univariate *F* tests were conducted to examine the differences in the adjustment variables analyzed, and, lastly, Bonferroni’s post-hoc test was applied.

## Results

### Distribution of the families in the parenting styles

Table 1 shows the families’ distribution according to their parenting style, as well as their means and standard deviations on each of the main dimensions of the classical model (acceptance/involvement and strictness/imposition). *A posteriori* analysis indicated that the main dimensions of the model were relatively orthogonal, *r*(586) = 0.11, *r*^2^ = 0.01, *p* < 0.01, and the cross-distribution of the families in relation to children’s sex, *χ*^2^(3) = 6.05, *p* > 0.05, and children’s age, *χ*^2^(18) = 17.43, *p* > 0.05, was statistically homogenous.

**Table 1:** Distribution of the families in the parenting styles, means (*M*), and standard deviations (*SD*) on acceptance/involvement and strictness/imposition.

	Authoritative	Indulgent	Authoritarian	Neglectful	Total
Frequency	145	138	117	186	586
Percentage	24.7	23.5	20.0	31.7	100
Acceptance/involvement					
*M*	3.54	3.44	2.71	2.70	3.09
*SD*	0.25	0.34	0.36	0.41	0.52
Strictness/imposition					
*M*	2.16	1.48	2.11	1.45	1.77
*SD*	0.29	0.22	0.32	0.20	0.42

Scores on acceptance/involvement and strictness/imposition ranged from 1 to 4.

### Multivariate analyses

The results obtained in the first MANOVA conducted with self-concept showed statistically significant differences in the main effects of parenting styles, Λ = 0.79, *F*(15, 1562.88) = 9.24, *p* < 0.001, and sex, Λ = 0.93, *F*(5, 566) = 8.67, *p* < 0.001. Statistically significant interaction effects were not obtained (α = 0.05).

The second MANOVA performed with empathy also showed statistically significant differences in the main effects of parenting styles, Λ = 0.89, *F*(12, 1500.43) = 5.43, *p* < 0.001, and sex, Λ = 0.91, *F*(4, 567) = 13.79, *p* < 0.001. Interaction effects were not statistically significant (α = 0.05).

### Univariate analyses of parenting and empathy

The univariate *F* tests performed with empathy showed statistically significant differences between parenting styles and the four dimensions of empathy assessed (see Table 2). Tests conducted *a posteriori* (Bonferroni, α = 0.05) indicated that adolescents from indulgent and authoritative families obtained higher scores on adopting perspectives, empathic stress, and empathic happiness than adolescents from authoritarian and neglectful families. Finally, adolescents from indulgent families scored higher on emotional understanding than adolescents from authoritarian and neglectful families.

**Table 2:** Means (standard deviations), *F* values, and Bonferroni’s test^#^ between parenting styles and cognitive and emotional empathy.

	Parenting style				*F*(3, 582)	Eta square
	Authoritative	Indulgent	Authoritarian	Neglectful		
Cognitive empathy						
Adopting perspectives	3.06^1^ (0.45)	3.17^1^ (0.48)	2.87^2^ (0.55)	2.92^2^ (0.47)	11.02***	0.054
Emotional understanding	2.96 (0.44)	3.03^1^ (0.50)	2.81^2^ (0.45)	2.84^2^ (0.46)	6.87***	0.034
Emotional empathy						
Empathic stress	2.64^1^ (0.57)	2.67^1^ (0.57)	2.46^2^ (0.51)	2.38^2^ (0.52)	10.42***	0.051
Empathic happiness	3.41^1^ (0.44)	3.42^1^ (0.46)	3.12^2^ (0.55)	3.21^2^ (0.48)	13.20***	0.064

1 > 2, ***p < 0.001

### Univariate analyses of parenting and self-concept

The results obtained on the univariate *F* tests showed statistically significant differences between parenting styles and the five dimensions of self-concept assessed (see Table 3). The Bonferroni tests (α = 0.05) indicated that adolescents from indulgent and authoritative families obtained higher scores on academic, social, family, and physical dimensions of self-concept than adolescents from authoritarian and neglectful families. In the emotional self-concept dimension, adolescents from indulgent families obtained better scores than adolescents from authoritative, authoritarian, and neglectful families.

**Table 3:** Means (standard deviations), *F* values, and Bonferroni’s test^#^ between parenting styles, self-esteem, and empathy.

	Parenting Style				*F*(3, 582)	Eta square
	Authoritative	Indulgent	Authoritarian	Neglectful		
Self-concept						
Academic	6.94^1^ (1.48)	7.02^1^ (1.41)	6.02^2^ (1.66)	6.37^2^ (1.58)	12.78***	0.062
Social	6.82^1^ (1.29)	7.21^1^ (1.24)	6.15^2^ (1.40)	6.35^2^ (1.54)	16.07***	0.077
Emotional	4.89^2^ (1.65)	5.56^1^ (1.57)	4.76^2^ (1.60)	5.07^2^ (1.66)	6.20***	0.031
Family	7.65^1^ (1.39)	7.91^1^ (1.05)	6.02^2^ (1.79)	6.94^2^ (1.53)	42.77***	0.181
Physical	6.16^1^ (1.62)	6.06^1^ (1.46)	5.27^2^ (1.70)	5.56^2^ (1.54)	9.47***	0.050

1 > 2, ***p < 0.001

### Univariate analyses of sex

Regarding empathy, females scored higher than males on the cognitive (adopting perspectives, emotional understanding) and emotional (empathic stress and empathic happiness) domains of empathy (see Table 4). With regard to the emotional and physical dimensions of self-concept, males obtained higher scores than females.

**Table 4:** Means (standard deviations), *F* values, and Bonferroni’s test^#^ between parenting styles, self-esteem, and empathy.

	Sex		*F*(1, 582)
	Males	Females	
*Cognitive empathy*			
Adopting perspectives	2.92 (0.50)	3.07 (0.48)	13.514***
Emotional understanding	2.85 (0.46)	2.95 (0.47)	6.54*
*Emotional empathy*			
Empathic stress	2.35 (0.52)	2.67 (0.55)	53.13***
Empathic happiness	3.19 (0.52)	3.37 (0.46)	18.16***
*Self-concept*			
Academic	6.47 (1.61)	6.70 (1.55)	2.88
Social	6.67 (1.52)	6.61 (1.37)	0.15
Emotional	5.34 (1.66)	4.88 (1.61)	11.46**
Family	7.15 (1.53)	7.17 (1.67)	0.02
Physical	6.07 (1.58)	5.53 (1.60)	16.60***

*p < 0.05, **p < 0.01. ***p < 0.001.

## Discussion

Given the important influence of parenting on children’s acquisition of a set of social values to develop capacity to take the perspective of others, and their self-regulatory abilities, as well as the relevant role of cultural influences and the mixed results reported in previous research, the main objective of this study was to examine which parenting style is related to the greatest adolescent cognitive and emotional empathy and self-concept. This study, therefore, contributes to the current international debate on optimal parenting, based on the theoretical model with four typologies.

The results confirmed main findings from previous studies. Adolescents from indulgent families reported equal or even better empathy and self-concept than those from authoritative families, whereas adolescents with non-warm households (those who define their parents as authoritarian and neglectful) reported worse scores. On cognitive empathy outcomes, adolescents from indulgent and authoritative families showed greater skill in adopting perspectives than their peers from authoritarian and neglectful homes. Indulgent parenting was related to the highest emotional understanding, whereas authoritarian and neglectful styles were related to the lowest emotional understanding. A similar tendency was found for the self-concept outcomes. Warm parenting (i.e., indulgent and authoritative) was constantly associated with better academic, social, emotional, family, and physical self-concept than non-warm parenting (i.e., authoritarian and neglectful). Additionally, in the emotional domain, adolescents from indulgent families obtained better self-concept scores than those with authoritative parents.

Classical studies in parenting literature usually consider parental demandingness as an essential component for an effective parental socialization. To achieve children with prosocial behaviors, in which empathy represents an essential component (i.e., understand and feel the others), the use of punishment and strictness is recommended to parents (Baumrind, 1983; Maccoby and Martin, 1983). Nevertheless, according to the present findings, strictness seems unnecessary or even detrimental for adolescent empathy and self-concept in line to some recent studies which suggested the benefits related to parental warmth, but not parental strictness (Perez-Gramaje et al., 2020; Queiroz et al., 2020; Gimenez-Serrano et al., 2022). Adolescents who achieve the greatest levels of empathy seem to benefit of parents that are warm and involved, which could be enough to achieve a greater orientation toward the others (i.e., empathy) and confidence in oneself (i.e., self-concept).

In the parenting literature, a main question is how parents might help their children to cope with social demands. To deal with these social demands, it is crucial for children and adolescents to acquire social values that allow them to regulate their actions, take the perspective of others, and develop self-regulatory abilities, including an adequate sense of self (Maccoby and Martin, 1983; Grusec and Goodnow, 1994; Grusec et al., 2017). The findings from the present study are consistent with some previous studies conducted in European (Rodrigues et al., 2013; Calafat et al., 2014; Garcia et al., 2019) and South-American countries (Martínez et al., 2007; Martínez and Garcia, 2008; Garcia et al., 2019), suggesting that the indulgent style (i.e., warmth but not strictness) is related to the best psychosocial competence and offers the same or even greater benefits than the authoritative style (i.e., warmth and strictness).

Since years scholars have discussed how parents can favor an effective socialization (Lewis, 1981; Baumrind, 1983). The cultural context could be crucial to examine why the same parenting based on the two main dimensions (i.e., warmth and strictness) is related to different benefits for children (Darling and Steinberg, 1993; Garcia et al., 2019; Pinquart and Gerke, 2019). Overall, the main parental component which help children seems to be strictness, especially useful when is accompanied by warmth (i.e., authoritative) as it was identified in classical studies with European-American middle-class families since seminal studies of (Baumrind, 1967; Baumrind and Black, 1967; Baumrind, 1971) and the following of Steinberg (Lamborn et al., 1991; Steinberg et al., 1994). Additionally, even parental strictness accompanied by lack of parental warmth could be benefit as for example in ethnic minorities from US (Baumrind, 1972; Chao, 2001) or Arab societies, (Dwairy and Achoui, 2006; Dwairy et al., 2006a). Nevertheless, in the digital society seems that, at least in some cultural settings, the main parental component of a successful socialization could be warmth, but not strictness (Garcia et al., 2019). The so-called third parenting stage (i.e., warmth without strictness) could favor adolescents internalize the social message transmitted by their parents.

Parents have as main responsibility raising children and transmit social values to them, although the parents used different strategies (based on warmth and strictness) and not all parents have the same success (Darling and Steinberg, 1993; Veiga et al., 2021; Sandoval-Obando et al., 2022). In fact, the literature has also examined the relationship between parenting patterns and the child psychosocial adjustment using different psychosocial adjustment criteria such as social anxiety (Gomez-Ortiz et al., 2019), cyberbullying (Gomez-Ortiz et al., 2018), resilience and attachment (Gomez-Ortiz et al., 2015). Effective socialization might be identified when adolescents have greater priority toward others (e.g., higher empathy in both cognitive and affective dimensions) and confidence in oneself (e.g., greater self-concept in different domains). Interestingly, and contrary to classical studies, at least in the so-called digital society, the main findings from the European country examined in the present study (i.e., Spain) showed that parental warmth seems to be the main parental component for an effective socialization, whereas strictness is not relevant or even could be detrimental: adolescents from families based on warmth without strictness (i.e., indulgent) reported equal or even more empathy and self-concept than those from homes based on warmth with strictness (i.e., authoritative).

Families based on warmth really achieve an effective socialization due to their adolescents report greater empathy and self-concept according to the present findings. It is possible that adolescents raised by warm parents benefit to an emotional climate based on reasoning, involvement, and acceptance, which could help them to internalize the societal values transmitted by their parents (confidence in the others and in oneself). On the opposite side, adolescents from non-warm homes (i.e., authoritarian and neglectful) could perceive their family is intrusive and not appreciated them, rejecting the message and societal values transmitted by their parents, including the importance considering others (cognitive and affective empathy) as well as the contribution of oneself as a valuable person for the society (self-concept). Regardless, for each specific cultural context is needed to examine which is the optimal parenting stage (Garcia et al., 2019).

Overall, the present findings do not agree with other previous studies examining the optimal parenting in various cultural contexts, highlighting the idea that the relationship between parenting and adolescent competence and adjustment might be different depending on the cultural and social context in which parental socialization take place (Darling and Steinberg, 1993; Pinquart, 2017; Pinquart and Kauser, 2018; Dakers and Guse, 2020; Sandoval-Obando et al., 2022). Both the indulgent and authoritative parenting styles are characterized by high warmth. However, only the authoritative style is also characterized by high parental strictness. Importantly, in other cultural contexts, high parental strictness is considered as the crucial parenting dimension defining optimal parenting as the authoritarian style (i.e., low parental warmth and high strictness), as in the case of some socialization outcomes in Asian and Middle Eastern societies (Chao, 2001; Dwairy et al., 2006b,c). Similarly, in other cultural contexts, parental strictness and high parental warmth are necessary in defining the authoritative style as leading to optimal adolescent competence and adjustment, mainly in Anglo-Saxon cultural contexts (Baumrind, 1971; Darling and Steinberg, 1993; Steinberg et al., 1994). By contrast, at least within the European cultural context examined (i.e., Spain), according to the present findings, parental strictness does not seem to be a crucial parenting dimension because with high parental warmth alone (high warmth and low strictness, the indulgent style), adolescents showed similar or even better empathic competence and self-concept than those whose parents are also characterized by high strictness (high warmth and high strictness, the authoritative style).

It seems that the cultural context in which parental socialization take place might vary the association between parenting and child adjustment (Darling and Steinberg, 1993; Pinquart and Kauser, 2018; Garcia et al., 2019). The same families (i.e., authoritative, indulgent, authoritarian, and neglectful) are living in different cultural contexts with variations in cultural values (e.g., vertical-horizontal, individualism–collectivism; Singelis et al., 1995; Martínez and Garcia, 2007). Parental warmth could be especially beneficial if the cultural context values the collective (e.g., the family), but with relationships between members that, despite having a different status (parents as adults vs. children and adolescents), tend to be more egalitarian and not so hierarchical, so the parental strict component (common in authoritative families) could be unnecessary or even detrimental since it could be perceived by the children as intrusive (Martínez and Garcia, 2007; Climent-Galarza et al., 2022). Thus, the same family (e.g., authoritarian, authoritative and indulgent) could have a different impact on child development, maybe because of the children’s assessment of whether their family loves and appreciates them (family self-concept; Baumrind, 1996; Deater-Deckard et al., 1996; Martínez et al., 2021), or perhaps in part by parenting beliefs (Ridao et al., 2021) which, in turn, are influenced by culture (Rubin and Chung, 2006; Sanchez et al., 2020). As a previous parenting study noted (Garcia et al., 2019), it seems that the three parenting stages (i.e., authoritarian, authoritative and indulgent) might coincide at the same time in different environments, context, and cultures, thus extending previous evidence on the traditional paradigm with only two stages (i.e., authoritarian and authoritative parenting styles). At least in the European cultural context examined (Spain), the third parenting stage (i.e., indulgent style) is again found to be the optimal parenting style.

It should be noted that previous evidence shows ambiguous findings about how the parent–child relationship could be associated with empathy outcomes (Boele et al., 2019). Most of this evidence captured parenting through isolated parental practices (e.g., reasoning, love-withdrawal, and power assertion), without analyzing it according to the two main orthogonal dimensions of parenting (i.e., warmth and strictness). However, this point is an important limitation, even though, as Hoffman highlighted (Hoffman and Saltzstein, 1967, p. 51), parents who use reasoning (induction) are not necessarily low on power assertion. Additionally, most previous studies do not take into account that parents help their children to develop empathic competence in different cultural contexts (Helwig et al., 2014; Boele et al., 2019).

The process of internalization of self-transcendence and conservation values involves socially-focused motivations (Sortheix and Schwartz, 2017), emphasizing the positive effects on others of fostering a child’s feelings of empathy and consideration for others (Hoffman, 1970; Lewis, 1981; Baumrind, 1983). Some previous studies that have examined the relationship between parenting styles (i.e., indulgent, authoritative, authoritarian, and neglectful) and the internalization of self-transcendence and conservation values in families Europe (Martínez and Garcia, 2007; Martinez et al., 2020), South America (Martínez et al., 2007), and more recently the United States (Garcia et al., 2019), also showed the benefits of the indulgent style (warmth but not strictness). Nevertheless, less is known about the relationship between parenting styles and empathy. In these previous studies, an important limitation is the way empathy is captured, through a global dimension (Garcia and Serra, 2019; Garcia et al., 2021). By contrast, the present study extends the benefits of indulgent parenting to four indicators of cognitive empathy (adopting perspectives and emotional understanding) and emotional empathy (empathic stress and empathic happiness), based on the classical theoretical framework distinguishing the emotional and cognitive features of empathy (Davis, 1983). In addition, the findings from this study make an important contribution by showing that the indulgent style is the optimal parenting style to foster self-concept, a socialization outcome that is still a source of debate in the parenting literature (Pinquart and Gerke, 2019).

In addition, in the present study there were no statistically significant interaction effects between parenting styles and sex neither between parenting styles and age. So, the relation between parenting styles and the patterns of adolescent self-concept and empathy are consistent across adolescent age and sex as in some previous studies (Riquelme et al., 2018; Perez-Gramaje et al., 2020; Queiroz et al., 2020), regardless the multiple differences that have been established in different aspects of developmental adjustment depending on age and sex, i.e., sex-and age-related differences, for example, females tend to report greater academic self-concept than males (Riquelme et al., 2018; Queiroz et al., 2020). According to the findings of the present study, adolescent daughters and sons achieve greater self-concept and empathy only when are raised by families that are warm, but not strict. Therefore, the benefits of the optimal parenting seem to transcend the boundaries of child sex and age in line with some previous studies (Riquelme et al., 2018; Queiroz et al., 2020). By contrast, evidence from present study does not support the idea that parents should be use different practices with their sons and daughters suggested by some gender scholars based on some previous studies (Miklikowska et al., 2011; Beckmann et al., 2021). For example, parenting need support (great warmth) predicted changes in empathic concern in daughters only (Miklikowska et al., 2011). Additionally, although it was not the main objective, sex-related differences in the present study agree with other previous studies carried out with adolescents (Garcia and Gracia, 2009; Veiga et al., 2015; Tur-Porcar et al., 2019; Musitu-Ferrer et al., 2019b). The highest empathic competence was found in females. Females reported greater empathy than males in both cognitive (adopting perspectives and emotional understanding) and emotional (empathic stress, and empathic happiness) domains. Males showed greater physical and emotional self-concept than females. It is important to note that there are different processes within and without the family that could affect the adolescent competence. For example, other family process such as marital satisfaction (Chis et al., 2022), parenting stress (Gomez-Ortiz et al., 2022), family functioning (Yeung, 2021) family climate (Hernandez-Serrano et al., 2021) and the predisposition to have more children (Gomez-Ortiz and Sanchez-Sanchez, 2022). The same is true for other influences not directly related to the family, for example, the individual child characteristics such as emotional intelligence (Cabello et al., 2021) or the cultural context (Sacca et al., 2021) or the historical time (e.g., digitalization; Hung, 2022).

The present study has strengths and some limitations. Regarding the strengths, it is necessary to highlight the following issues: (i) This study was carried out taking into account the minimum sample size required to achieve adequate statistical power, thus reducing the likelihood of making type II errors in statistical inference and increasing the likelihood of detecting real relationships between parenting styles and children’s psychological adjustment. (ii) Parenting styles were measured using an orthogonal instrument based on the classical two-dimensional and four-typology theoretical model of parental socialization. (iii) Findings from the present study are easily replicable by other researchers all over the world, contributing to the current international debate about optimal parenting. (iv) Adolescent empathy is captured with four indicators, distinguishing between cognitive (adopting perspectives and emotional understanding) and emotional (empathic stress and empathic happiness) features of interpersonal empathy, as well as the self-concept competence, captured with five indicators (academic, social, emotional, family, and physical). Empathy is usually examined in parenting studies focused on early and middle childhood (e.g., Hoffman, 1970), but less in adolescence as in the present study.

As a limitation, it should be noted that the classification of the families in one of the four typologies of the model was based on the children’s ratings, and one might think that parents’ responses would offer more objective information. However, some studies obtained the same findings with different parent response reports (Yeung et al., 2019). Furthermore, some empirical evidence shows that parents’ responses tend to be more biased by social desirability (Barry et al., 2008). Another limitation that is common in this research area refers to the cross-sectional design of the study. Thus, it is not possible to draw conclusions about causal relationships between the studied variables.

Longitudinal studies aim to test if there are continuing benefit or harming of particular patterns to parenting (e.g., indulgent, authoritative, authoritarian and neglectful) in specific developmental periods (e.g., adolescence). Overall, the parenting style identified as the optimal is usually related to the greater psychosocial competence over time (Steinberg et al., 1994; Milevsky, 2020). Although previous longitudinal studies confirmed the long-term impact of parenting on child empathy and self-concept (Koestner et al., 1990; Miklikowska et al., 2011; Yoo et al., 2013; Pinquart and Gerke, 2019), some of them examined different parenting practices, but not in the general context of parenting styles (e.g., Yoo et al., 2013), so results are difficult to compare with other studies, or even the findings revealed that the same parenting practice (e.g., need-supportive parenting) had a different impact on sons and daughters when is used by mothers or fathers (see Miklikowska et al., 2011). In this regard, further research using longitudinal designs is needed in order to analyze these relationships in depth (Garcia and Gracia, 2009). Future studies should examine, at the same time, the impact of parents in the internalization of social values, empathy and prosocial behaviors or self-concept to capture more accurately an effective socialization, but present study offer clearly evidence about how parents could contribute (positively, but also negatively) to adolescent empathy and self-concept.

Despite these limitations, the results of this study contribute to the validation of the classical theoretical model of parental socialization across the globe (Garcia et al., 2019; Pinquart and Gerke, 2019), adding empirical evidence about the benefits of the so-called third parenting stage, the indulgent style (Garcia et al., 2019). Therefore, parenting practices pertaining to the acceptance/involvement dimension, such as parental warmth and support and the use of dialog and reasoning with children, should be taken into account in prevention and intervention programs for improving parent–child relationships and fostering children’s adjustment in the family context and in society.

## Data availability statement

The raw data supporting the conclusions of this article will be made available by the authors via email request to the corresponding author, without any undue reservation.

## Ethics statement

The studies involving human participants were reviewed and approved by College Research Ethics Committee (CREC) of Nottingham Trent University (protocol code no. 2017/90, May 2017). Written informed consent to participate in this study was provided by the participants’ legal guardian/next of kin.

## Author contributions

MF, OG, MA, RG-R, and FG contributed to conception and design of the study. MF, MA, and FG organized the database and performed the statistical analysis. MF and OG wrote the first draft of the manuscript. MA, RG-R, and FG wrote sections of the manuscript. All authors contributed to manuscript revision, read, and approved the submitted version.

## Funding

The research reported in this study has been supported by grant CIAICO/2021/252 (Generalitat Valenciana and European Regional Development Fund), FPU20/06307 (Ministry of Universities, Government of Spain), ACIF/2016/431 and BEFPI/2017/058, which provided funding for a research stay at the Nottingham Trent University, United Kingdom (Generalitat Valenciana and European Social Fund).

## Conflict of interest

The authors declare that the research was conducted in the absence of any commercial or financial relationships that could be construed as a potential conflict of interest.

## Publisher’s note

All claims expressed in this article are solely those of the authors and do not necessarily represent those of their affiliated organizations, or those of the publisher, the editors and the reviewers. Any product that may be evaluated in this article, or claim that may be made by its manufacturer, is not guaranteed or endorsed by the publisher.
